# HTLV-1 Tax binds to and stabilizes the SUMO-targeted ubiquitin ligase RNF4 during DNA damage response

**DOI:** 10.1186/1742-4690-11-S1-P98

**Published:** 2014-01-07

**Authors:** Xin Guo, Andrea Baillo, Sucharita M Dutta, Oliver Kerscher, O John Semmes

**Affiliations:** 1 Department of Microbiology and Molecular Cell Biology, Eastern Virginia Medical School, Norfolk, VA, USA; 2 The Leroy T. Canoles Jr Cancer Research Center, Eastern Virginia Medical School, Norfolk, VA, USA; 3 Department of Biology, College of William and Mary, Williamsburg, VA, USA

## 

Human T-cell Leukemia Virus type 1 Tax protein has been ascribed numerous activities and is clearly functionally pleiotropic with respect to virus biology. In fact, we previously reported that RNF4 regulates the subcellular localization of Tax, establishing this STUbL as a key factor in the compartmentalization of Tax-mediated functions. One of the activities of Tax is to promote genomic instability, a function that we have proposed derives from the competitive sequestration of cellular damage response proteins. Recent studies revealed that RNF4 was required for DNA break repair by regulating ubiquitylation and recycling of DNA damage response (DDR) proteins. Because we had already established that Tax bound to RNF4, we examined whether RNF4 mediates Tax-induced genomic instability. Tax binding to RNF4 is mediated via Tax amino acids 202-253. Exogenous cellular expression of Tax markedly increased steady-state levels of RNF4 protein without affecting its mRNA level, while the deletion mutant TaxD202-253 showed no detectable effect. In the presence of cycloheximide, RNF4 displayed a basal half-life of approximately 12-16 hours. In response to DNA damage, RNF4 was stabilized with a two-fold increase in half-life. This result indicates a role for protein stability in the cellular damage response. Interestingly, co-expression of Tax inhibited the DNA damage-induced stabilization of RNF4, suggesting that Tax impairs RNF4 function. We are currently examining the potential effects of Tax on RNF4 protein post-translation modification (PTM), such as phosphorylation, ubiquitylation and sumoylation during DNA damage response with both proteomic and biochemical techniques.

